# Longitudinal association between handgrip strength and depressive symptoms in middle-aged and older Chinese adults: mediating role of functional limitation

**DOI:** 10.3389/fpubh.2025.1496641

**Published:** 2025-02-18

**Authors:** Yanchang Liu, Junling Cui, Xin Luo, Zhuzhu Wang, Ziyi Shen, Yan Fang, Chengcheng Li, Jingfang Hong

**Affiliations:** School of Nursing, Anhui Medical University, Hefei, China

**Keywords:** depressive symptoms, handgrip strength, functional limitation, middle-aged and older adults, mediation analysis, China, CHARLS

## Abstract

**Background:**

The relationship between handgrip strength (HGS) at baseline and subsequent depressive symptoms among middle-aged and older Chinese adults remains highly uncertain. This research endeavored to investigate the effect of functional limitation on the association between these two variables.

**Methods:**

A total of 5,684 participants aged ≥45 years from the China Longitudinal Study of Health and Retirement (CHARLS) were enrolled, using data from the 2015 and 2018 waves. Functional limitation was evaluated based on participants’ self-reported basic activities of daily living (BADL) and instrumental activities of daily living (IADL). Logistic regression models were utilized to analyze the relationship between HGS and subsequent depressive symptoms, and bootstrap analysis was performed to explore the potential mediating role of functional limitation.

**Results:**

After adjusting for confounders, an inverse correlation was detected between HGS and functional limitation (*B* = -0.885, *p* < 0.001), a positive correlation was found between functional limitation and subsequent depressive symptoms (*B* = 1.054, *p* < 0.001). The mediated effect size of HGS on depressive symptoms through functional limitation was a*b = −0.933 (BCa 95% CI: −1.224, −0.642). Functional limitation had a significant impact on 18.9% of the overall association.

**Conclusion:**

Among the Chinese middle-aged and senior population, functional limitation accounted for 18.9% of the connection between HGS and depressive symptoms. Interventions targeting the enhancement of muscle strength should be regarded as crucial elements for maintaining physical function and preventing depressive symptoms.

## 1 Introduction

With the intensification of population aging, mental health problems are becoming increasingly prominent among the older adult population. Approximately 14% of adults aged 60 and above are suffering from mental disorders, including anxiety, depression, and feelings of loneliness (1). Relatively speaking, among these negative emotions, it is commonly acknowledged that depression is a prominent psychological dysfunction and has emerged as a crucial public health concern. In 2021, the WHO reported that approximately 280 million individuals globally suffered from depression, which is a principal contributor to disability and the worldwide disease burden (2). In China, a considerable part of the middle-aged and senior citizens exhibit depressive symptoms. A meta-analysis has indicated a prevalence rate of up to 22.7% (3, 4), thereby impacting their life quality and increasing healthcare costs (5). Additionally, depressive symptoms are associated with disabilities, elevated mortality rates, and an increased probability of comorbidities (6). The bio-psycho-social medical model emphasizes the complex interconnection among physical conditions, mental well-being, and social adaptability (7). Consequently, exploring the underlying risk elements and preventive strategies for depressive symptoms from the perspective of physical function is crucial for enhancing the physical and mental well-being of middle-aged and older individuals.

Handgrip strength (HGS) is commonly employed to assess the overall muscular strength (8). A range of unfavorable health consequences, such as functional disabilities, chronic diseases, and all-cause mortality, have been associated with low HGS (9). Moreover, HGS has been linked to psychological issues. Multiple studies from diverse countries have demonstrated that greater HGS is correlated with a reduced likelihood of depressive symptoms in middle-aged and senior populations (10–15). Additionally, a meta-analysis involving 30,727 participants corroborated a negative association (16). A number of cohort investigations have further examined the relationship between HGS and the risk of depressive symptoms in middle-aged and older individuals (aged ≥50 years). These studies have identified a connection between lower baseline HGS and a higher incidence of subsequent depressive symptoms (17–22), which provides a basis for investigating the causal connection between these two elements. However, prior research has not comprehensively elucidated the mediating mechanisms by which HGS influences depressive symptoms.

Functional limitation encompasses both basic activities of daily living (BADL) and instrumental activities of daily living (IADL). BADL pertains to physical self-care abilities, including dressing and eating. In addition, IADL refers to the more advanced skills that older individuals need to live independently in their communities, such as shopping and cooking (23). It was indicated that functional restrictions might trigger a decline in self-reliance and self-governance (24). Diminished physical capabilities can result in functional impairment and an elevated susceptibility to incidents like falls, necessitating hospitalization, and even leading to mortality (25), which often places a significant burden on the health-related quality of life for middle-aged and senior individuals. Additionally, the age-driven reductions in muscle strength and muscle mass substantially contribute to functional limitation in older individuals (26). Sufficient muscular mass and strength are essential for performing activities of daily living (ADL), as they serve as indicators of body function and comprehensive quality of life (27). HGS is widely utilized as a muscle strength test due to its simplicity and cost-effectiveness. Previous studies have revealed that low HGS is positively correlated with functional limitation in middle-aged and older adult individuals (23, 26, 28), suggesting that maintaining muscle strength may be a crucial means of slowing the progression of dysfunction. Simultaneously, an increasing body of research also indicates a relationship between decreased daily activity performance and depressive symptoms in older adults (29–31). Tian et al. discovered that those individuals afflicted with severe ADL disabilities were subsequently at an heightened risk of developing depressive symptoms based on a longitudinal group-based trajectory model (32). The possible mechanism implies that functional disability can be regarded as a stressful condition that may harm mental health and raise the likelihood of depressive symptoms over an extended period (33).

Most importantly, the activity restriction model has demonstrated that restrictions in normal activities act as forecasters of depressive symptoms and may mediate the connection between illness and depressive symptoms (34). Accordingly, we have hypothesized that one of the latent mechanisms underlying the connection between HGS and depressive symptoms might be via functional limitation. Nevertheless, the degree to which and the manner in which functional limitation impacts the association between HGS and depressive symptoms among middle-aged and older adults remain unclear.

In summary, no studies have probed the underlying potential intermediary mechanisms within the longitudinal relationship between HGS and the risk of depressive symptoms among middle-aged and older adults. Consequently, the objective of our research was to examine the correlation between HGS and the occurrence of depressive symptoms in Chinese people aged 45 and older, as well as to examine the mediating role that functional limitation plays in this association.

## 2 Methods

### 2.1 Participants

**Figure 1:**
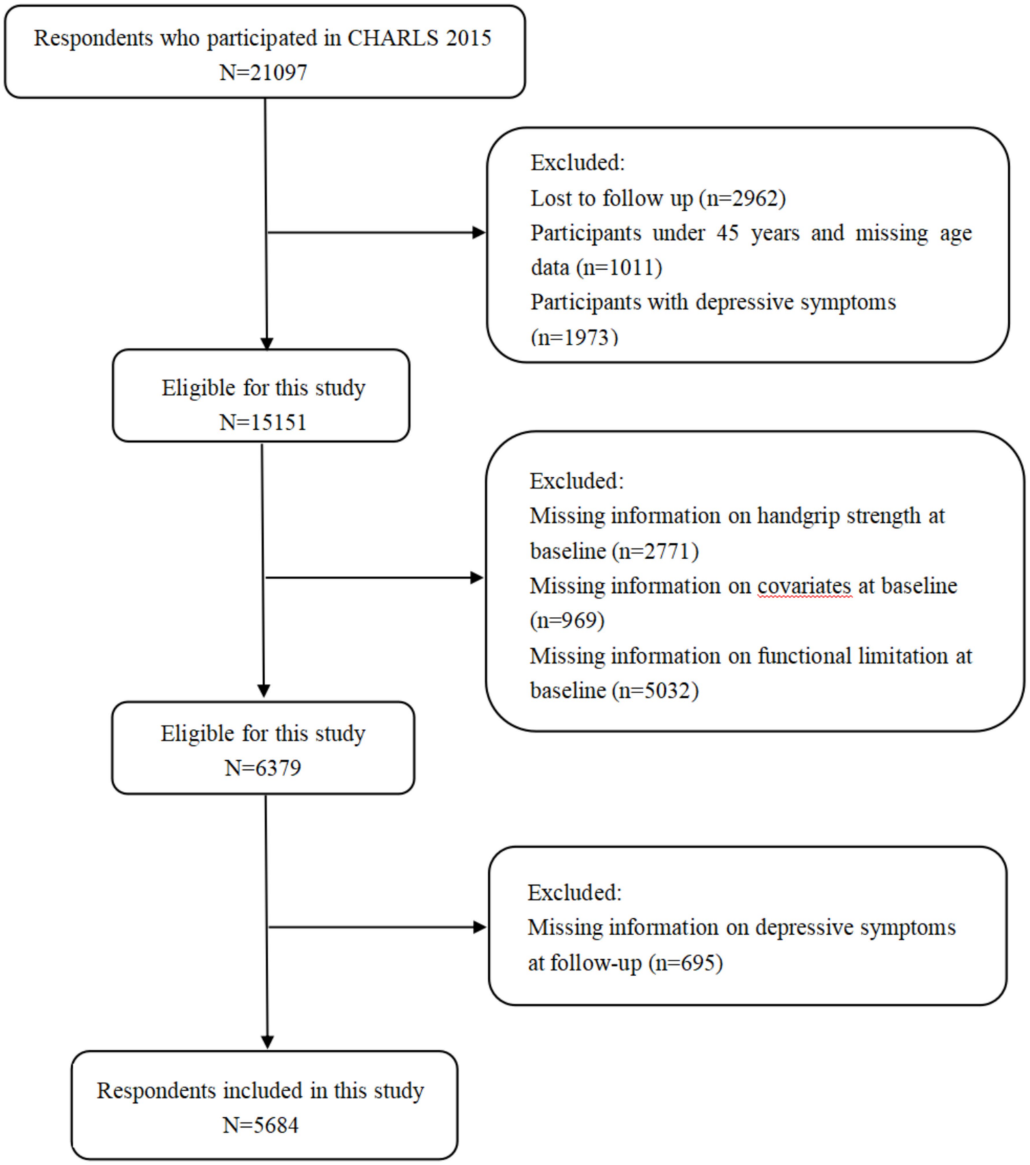
Flowchart of participant selection.

The CHARLS (China Health and Retirement Longitudinal Study), an annual countrywide research conducted over four phases, accumulates data on health and wellness from people aged 45 or more in the middle-aged and senior group. The general public’s sample was chosen using a method involving multiple phases of probabilistic sampling. Comprehensive methods for CHARLS sampling can be found in other publications (35). Using Wave 3 (2015) as a baseline, our survey encompassed 21,097 participants from 12,235 households, of which 18,135 took part in a subsequent study executed 3 years later. In the present study, the exclusion criteria for participants were as follows: (1) being younger than 45 years old; (2) having no functional limitation data; (3) lacking CESD-10 scale data; (4) having no HGS data; and (5) missing values in covariates data. Finally, a total of *N* = 5684 participants were included in the analysis (Figure 1).

### 2.2 Measurement

#### 2.2.1 Handgrip strength

In 2015, the assessment of HGS was carried out by operating a hydraulic handgrip dynamometer. The participants were directed to stand upright, with their arms loosely bent rather than close to their bodies. Subsequently, they were required to exert maximum force to compress the dynamometer, performing the procedure twice for each hand. The final result was obtained by taking the average value of the measurements from both the right and left hands. To minimize the potential influence of body weight on the calculation of handgrip power, the relative HGS index was calculated as handgrip strength (kg)/body weight (kg). The individuals were categorized into quartiles based on their relative HGS indexes, and those in the bottom quartile were classified as having weaker HGS (36).

#### 2.2.2 Depressive symptoms

In 2018, the evaluation involved the collection of measurements of depressive symptoms using the CESD-10 scale. This instrument exhibited considerable accuracy in evaluating depressive symptoms in Chinese adults (37). There are 10 items, each with 4 choices from last week: (1) little or no time (<1 day); (2) not much time (1–2 days); (3) sometimes or half of the time (3–4 days); and (4) most or all of the time (5–7 days). The scoring scale varies between 0 and 30, signifying increased severity of depressive symptoms in higher scores (38). In our study, a cutoff of 12 points was set to differentiate between participants who had depressive symptoms and those who did not.

#### 2.2.3 Functional limitation

In 2015, the BADL and IADL scales were utilized to assess functional limitation in middle-aged and older adults. The BADL scale encompasses six tasks, including dressing, eating, bathing, going to bed, using the toilet, and controlling urination (39). The IADL scale comprises five key aspects: housework, cooking, shopping, handling money management, and taking medication (40). All items have four options: “do it without difficulty,” “do it but with difficulty,” “do it with difficulty and need help,” and “cannot do it.” 11 items score between 11 and 44, where elevated scores signify increased functional limitation (41). Prior research has demonstrated a hierarchical interrelationship between IADL and BADL across a spectrum (42). The categorization of functional disability patterns was delineated as follows: (1) no limitation; (2) only IADL limitation; (3) only BADL limitation; and (4) both IADL and BADL limitations.

#### 2.2.4 Covariates

The covariates encompassed socio-demographic features (such as age, gender, education, marital status, residence, and working status); additionally, health behavior (smoking and drinking), physical activity, body mass index (BMI), and chronic disease were also considered. Education was classified as primary school or below, middle school, high school or above. Marital status was categorized as married and cohabiting, married and separated, divorced or widowed. Residence was divided into rural village and urban community. Working status was classified as retired or working. Categories for smoking and drinking status were classified as never, former, or current; physical activity was categorized as none, light, moderate, or vigorous; BMI was classified into four categories: underweight (BMI < 18.5), normal (18.5 ≤ BMI < 24.0), overweight (24.0 ≤ BMI < 28.0), and obesity (BMI ≥ 28.0). The number of chronic diseases was assessed by 14 self-reported non-communicable diseases, including hypertension, chronic lung disease, diabetes, cancer, stroke and so on and the total count of chronic diseases was summed up as no chronic disease, one chronic disease, two chronic diseases, or multimorbidity. The possible confounding factors were evaluated at baseline.

### 2.3 Statistical analysis

The statistical evaluation was executed through the application of Stata 17.0 and IBM SPSS 27.0. First, data were presented as means and standard deviations (SD) for normally distributed continuous variables and as medians and quartiles for variables that were not normally distributed, and frequencies and percentages were used to describe categorical variables. To compare the baseline characteristics of the two groups (non-depressive symptoms and depressive symptoms), the *t*-test was used for continuous variables and the *χ^2^* test was used for categorical variables. Second, logistic regression model was used to analyze the relationship between HGS and depressive symptoms. Third, we applied the Pearson and Spearman’s correlation analysis to estimate the association among the principal variables. Finally, a mediation model proposed by Baron and Kenny (43) was used to examine the mediating effect of baseline functional limitation on the relationship of baseline HGS on depressive symptoms that emerged during the follow-up period, and a non-parametric bootstrap method with 1,000 repeated samplings was adopted to evaluate the total, indirect, and direct effects (44). For all regression analyses, unstandardized regression coefficients (*B*) with corresponding standard errors were reported. Standardized regression coefficients (*β*) were also reported as variables under study were measured on different scales. The mediation effect is considered to be statistically significant if 0 falls outside the bias-corrected and accelerated 95% confidence interval (95% CI) of each path coefficient. Participants with cancer, chronic lung disease and stroke were excluded and sensitivity analyses were carried out to assess the robustness of the results. The reported confidence interval was calculated at the 95% level and statistical significance was considered as a two-tailed *p*-value <0.05.

### 2.4 Ethical approval

The CHARLS was approved by the Biomedical Ethics Review Committee of Peking University (IRB00001052-11015), and participants gave written informed consent before participating in the interview.

## 3 Results

### 3.1 Characteristics of the participants

The essential attributes of the participants are outlined in Table 1. 1,527 participants (26.86%) described experiencing depressive symptoms throughout the 2,018 follow-up period. In comparison to individuals without depressive symptoms, those exhibiting this feeling tended to be female (62.48%), had a lower education level (78.32%), urban (71.05%), and had multimorbidity (36.54%). Furthermore, they had relatively lower HGS and higher functional limitation.

**Table 1:** Participant characteristics categorized based on depressive symptoms at follow-up (*N* = 5,684).

Characteristics	Depressive symptoms			*p* value
	No	Yes	Total	
No. of subjects	4,157 (73.14)	1,527 (26.86)	5,684	
Age, mean (SD)	59.59 (9.10)	60.03 (9.05)	59.71 (9.08)	0.101
Gender				**<0.001**
Male	2,171 (52.23)	573 (37.52)	2,744 (48.28)	
Female	1986 (47.77)	954 (62.48)	2,940 (51.72)	
Education				**<0.001**
Primary school or below	2,735 (65.79)	1,196 (78.32)	3,931 (69.16)	
Middle school	913 (21.96)	232 (15.19)	1,145 (20.14)	
High school or above	509 (12.24)	99 (6.48)	608 (10.70)	
Marital status				**0.041**
Married and cohabiting	3,576 (86.04)	1,253 (82.06)	4,829 (84.97)	
Married and separated	195 (4.69)	74 (4.85)	269 (4.73)	
Divorced or widowed	385 (9.26)	200 (13.10)	585 (10.29)	
Residence				**<0.001**
Rural village	1,631 (39.24)	442 (28.95)	2073 (36.47)	
Urban community	2,526 (60.76)	1,085 (71.05)	3,611 (63.53)	
Working status				**0.002**
Working	2,999 (72.14)	1,111 (72.76)	4,110 (72.31)	
Retired	1,158 (27.86)	416 (27.24)	1,574 (27.69)	
Smoking status				0.618
Never	2,208 (53.12)	961 (62.93)	3,169 (55.75)	
Former	680 (16.36)	202 (13.23)	882 (15.52)	
Current	1,269 (30.53)	364 (23.84)	1,633 (28.73)	
Drinking status				0.201
Never	2,105 (50.64)	908 (59.46)	3,013 (53.01)	
Former	441 (10.61)	161 (10.54)	602 (10.59)	
Current	1,611 (38.75)	458 (29.99)	2069 (36.40)	
Physical activity				**0.036**
None	362 (8.71)	142 (9.30)	504 (8.87)	
Light	1,262 (30.36)	424 (27.77)	1,686 (29.66)	
Moderate	1,575 (37.89)	563 (36.87)	2,138 (37.61)	
Vigorous	958 (37.89)	398 (26.06)	1,356 (23.86)	
Chronic disease				**<0.001**
0	1,271 (30.57)	310 (20.3)	1,581 (27.81)	
1	1,080 (25.98)	322 (21.09)	1,402 (24.67)	
2	825 (19.85)	337 (22.07)	1,162 (20.44)	
≥3	981 (23.6)	558 (36.54)	1,539 (27.08)	
Body mass index				**0.003**
Underweight	186 (4.47)	94 (6.16)	280 (4.93)	
Normal	1911 (45.97)	740 (48.46)	2,651 (46.64)	
Overweight	1,472 (35.41)	476 (31.17)	1948 (34.27)	
Obesity	588 (14.14)	217 (14.21)	805 (14.16)	
Handgrip strength, mean (SD)	0.51 (0.14)	0.46 (0.15)	0.49 (0.15)	**<0.001**
Functional limitation				**<0.001**
No limitation	3,198 (76.93)	894 (58.55)	4,092 (71.99)	
BADL limitation	310 (7.46)	158 (10.35)	468 (8.23)	
IADL limitation	423 (10.18)	221 (14.47)	644 (11.33)	
IADL and BADL limitations	226 (5.44)	254 (16.63)	480 (8.44)	

The bolded values indicate that *p* < 0.05, which is statistically significant.

### 3.2 The correlation between handgrip strength at baseline and subsequent depressive symptoms

Binary logistic regression was used to examine the association between HGS and depressive symptoms. The results in Table 2 suggested that higher levels of HGS are associated with a lower risk of developing depressive symptoms. After controlling the confounding variables (Model 3), compared with Quartile 1, the OR values of HGS in the emergence of depressive symptoms were 0.72 (0.61, 0.85), 0.62 (0.52, 0.75), 0.53 (0.43, 0.66), respectively.

**Table 2:** The relationship between handgrip strength at baseline and subsequent depressive symptoms.

Logistic regression models	Quartile of handgrip strength per body weight (kg/kg)				
	Quartile 1	Quartile 2	Quartile 3	Quartile 4	*p* value
No. of depressive symptoms (CES-D ≥ 12)	509	395	334	289	
Crude model	Reference	0.69 (0.56, 0.81)^a^	0.55 (0.47, 0.65)	0.46 (0.39, 0.54)	<0.001
Adjusted model 1^b^	Reference	0.70 (0.59, 0.82)	0.58 (0.49, 0.70)	0.50 (0.40, 0.62)	<0.001
Adjusted model 2^c^	Reference	0.74 (0.63, 0.87)	0.65 (0.55, 0.77)	0.58 (0.48, 0.70)	<0.001
Adjusted model 3^d^	Reference	0.72 (0.61, 0.85)	0.62 (0.52, 0.75)	0.53 (0.43, 0.66)	<0.001

a Odds ratio (95% CI) (all such values).

b Adjusted for age, gender and BMI.

c Additionally adjusted for smoking, drinking, education, residence.

d Based on model 2, further adjusted for chronic disease, physical activity, working status.

### 3.3 Correlations among key variables

Individuals with depressive symptoms were excluded from the baseline analysis. Spearman correlation analysis and Pearson correlation analysis demonstrated an inverse link between the initial HGS and future depressive symptoms (*r* = −0.161, *p* < 0.001). A reverse correlation was observed between functional limitation and HGS (*r* = −0.207, *p* < 0.001). While a positive link was present with later depressive symptoms (*r* = 0.219, *p* < 0.001) as illustrated in Table 3.

**Table 3:** Correlations among depressive symptoms, handgrip strength, and functional limitation.

Variables	Depressive symptoms	Handgrip strength	Functional limitation
Depressive symptoms	1.000		
Handgrip strength	−0.161***	1.000	
Functional limitation	0.219***	−0.207***	1.000

*** *p*-value < 0.001.

### 3.4 The intermediary impact of functional limitation on how initial HGS correlates with subsequent depressive symptoms

Table 4 revealed the mediating role of functional limitation, following the adjustment for control variables (*β* = 0.17, *p* < 0.001). We executed a regression model analysis with adjustments, including a mediation effect model achieved by controlling for age, gender, marital status, education, residence, drinking status, working status, BMI, physical activity and chronic disease. Model 1 exhibited that middle-aged and older adults with HGS in Quartile 1 had a significantly higher level of depressive symptoms compared with those in Quartile 4 at baseline (*β* = 0.12, *p* < 0.001). After including the mediator of functional limitation in Model 2, the direct effect of HGS at baseline on depressive symptoms at follow-up was still significant (*β =* 0.10, *p* < 0.001).

**Table 4:** The mediating effect of functional limitation on the relationship between handgrip strength at baseline and subsequent depressive symptoms in middle-aged and older adult people (*N* = 5,684).

	Model 1				Model 2			
	*B*	*SE*	*β*	*p* value	*B*	*SE*	*β*	*p* value
Handgrip strength								
Quartile 4								
Quartile 1	1.71	0.28	0.12	**<0.001**	1.36	0.28	0.10	**<0.001**
Quartile 2	0.89	0.25	0.06	**<0.001**	0.78	0.25	0.06	**0.002**
Quartile 3	0.49	0.23	0.04	**0.034**	0.42	0.23	0.03	0.063
Gender								
Male								
Female	1.06	0.27	0.09	**<0.001**	0.92	0.26	0.08	**<0.001**
Age	−0.05	0.01	−0.07	**<0.001**	−0.06	0.01	−0.10	**<0.001**
Education								
Primary school or below								
Middle school	−0.99	0.20	−0.07	**<0.001**	−0.77	0.20	−0.05	**<0.001**
High school or above	−1.62	0.26	−0.08	**0.001**	−1.34	0.26	−0.07	**<0.001**
Marital status								
Married and cohabiting								
Married and separated	−4.06	5.79	−0.14	0.484	−1.14	5.70	−0.04	0.841
Divorced or widowed	−3.18	5.78	−0.16	0.582	−0.37	5.69	−0.02	0.948
Residence								
Rural village								
Urban community	1.05	0.17	0.08	**<0.001**	0.88	0.17	0.07	**<0.001**
Working status								
Retired								
Working	0.10	0.20	0.01	0.627	0.20	0.19	0.02	0.309
Smoking status								
Never								
Former	0.08	0.28	0.01	0.776	0.03	0.28	0.00	0.915
Current	0.23	0.25	0.02	0.357	0.21	0.25	0.02	0.405
Drinking status								
Current								
Never	0.36	0.19	0.03	0.063	0.35	0.19	0.03	0.065
Former	0.09	0.27	0.01	0.735	−0.04	0.27	0.00	0.886
Physical activity								
Light								
None	0.34	0.29	0.02	0.246	0.28	0.29	0.01	0.338
Moderate	0.20	0.19	0.02	0.287	0.33	0.19	0.03	0.080
Vigorous	0.73	0.22	0.05	**0.001**	0.73	0.22	0.05	**0.001**
Chronic disease								
0								
1	0.77	0.22	0.06	**<0.001**	0.70	0.21	0.05	**0.001**
2	1.54	0.23	0.10	**<0.001**	1.44	0.23	0.10	**<0.001**
≥3	2.91	0.22	0.21	**<0.001**	2.49	0.22	0.18	**<0.001**
Body mass index								
Normal								
Underweight	0.82	0.37	0.03	**0.025**	0.59	0.36	0.02	0.104
Overweight	−0.91	0.18	−0.07	**<0.001**	−0.94	0.18	−0.07	**<0.001**
Obesity	−1.23	0.25	−0.07	**<0.001**	−1.20	0.25	−0.07	**<0.001**
Functional limitation								
No limitation								
BADL limitation					1.67	0.28	0.08	**<0.001**
IADL limitation					1.34	0.25	0.07	**<0.001**
IADL and BADL limitations					3.71	0.29	0.17	**<0.001**
F	24.37				29.18			
Adjusted R-squared	0.093				0.122			

The bolded values indicate that *p* < 0.05, which is statistically significant.

**Figure 2:**
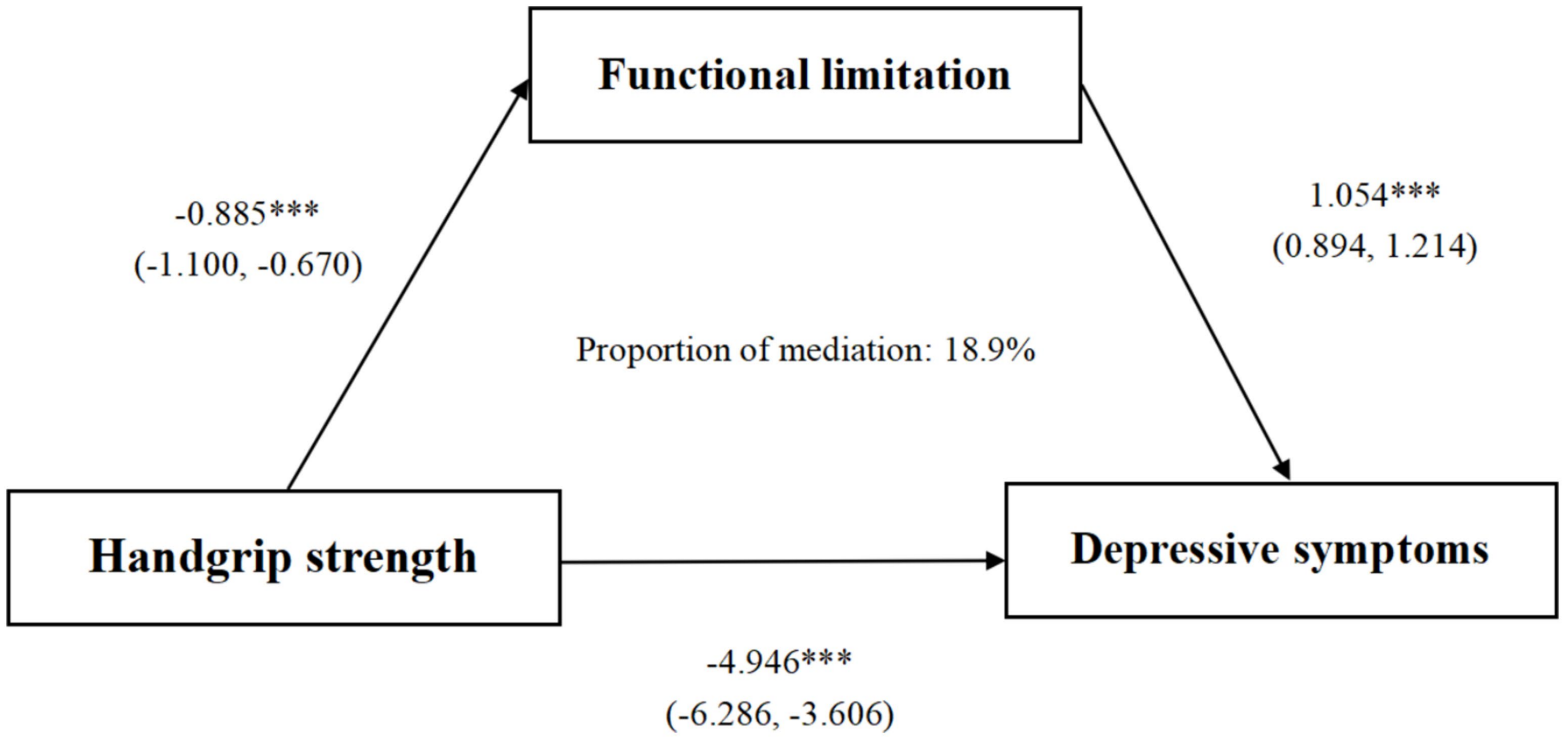
Path diagram of the association between functional limitation and depressive symptoms as a mediator. *** *p* < 0.001. Gender, age, education, marital status, residence, drinking status, smoking status, chronic disease, BMI, physical activity, and working status were controlled.

The bootstrap method (with 1,000 repeated samplings) was used to examine the mediating effect of functional limitation between HGS and depressive symptoms. Table 5 demonstrated that the direct effect was −4.013 (Bootstrap 95% CI: −5.414, −2.611). Meanwhile, the indirect effect of HGS on depressive symptoms through functional limitation was −0.933 (Bootstrap 95% CI: −1.224, −0.642). Figure 2 showed that functional limitation was a key factor linking HGS and depressive symptoms, explaining 18.9% of the total variation. That is, the occurrence of depressive symptoms among middle-aged and older adults with low HGS may be partially caused by functional limitation.

**Table 5:** Mediating effect of functional limitation on handgrip strength at baseline and subsequent depressive symptoms.

	Effect	SE	*t*	*p*	95%CI	
					Lower confidence interval	Upper confidence interval
Total effect	−4.946	0.684	−7.235	<0.001	−6.286	−3.606
Direct effect	−4.013	0.715	−7.163	<0.001	−5.414	−2.611
Indirect effect	−0.933	0.148^a^	–	<0.001	−1.224^b^	−0.642^c^

SE, standard error.

a Bootstrap standard error.

b BootLow confidence interval.

c BootUpper confidence interval.

### 3.5 Sensitivity analysis

After excluding individuals with cancer, chronic lung disease, and stroke, the outcomes remained largely unchanged. The overall impact of HGS on depressive symptoms was −4.878 (95% CI: −6.358, −3.398). Indirect mediation via functional limitation was −0.776 (95% CI: −1.054, −0.498). The indirect effects were significant because the null values were outside the 95% CI (Supplementary Figure S1; Supplementary Tables S1, S2).

## 4 Discussion

This study represents the first exploration to investigate how functional limitation acts as a mediator in the connection between HGS and depressive symptoms, specifically within a population-based cohort of middle-aged and older Chinese adults. The findings of this study demonstrate that baseline HGS was significantly correlated with the occurrence of depressive symptoms during the three-year follow-up period. In line with our presumptions, the connection between HGS and later-occurring depressive symptoms was partly mediated by functional limitation.

It was observed that high HGS levels at baseline were correlated with diminished incidence of depressive symptoms among middle-aged and older persons. Given the significant covariance between strength capacity and body mass, we opted to use a normalized HGS value relative to weight to explore the link between muscle strength and depressive symptoms. It was uncovered that with Quartile 1 (the lowest level of HGS) as the control group, the hazard of depressive symptoms was progressively reduced in the other three groups. Previously, in cross-sectional studies, it was reported that there were inverse relationships between HGS and depressive symptoms (10, 11, 45, 46). However, it is difficult to ascertain a potential causal relationship based on cross-sectional data. Merely three cohort studies have investigated whether HGS can forecast the onset of depressive symptoms in middle-aged and older Chinese populations (19, 22, 47). One study that utilized a normalized HGS value by weight discovered that the vulnerability of depressive symptoms declined steadily with increasing weighted and absolute HGS after multivariate adjustments during the follow-up period, which is consistent with the results of our study.

Our results were consistent with those of previous studies, which indicated that a decline in HGS was associated with a reduced likelihood of functional limitation (26, 48). HGS serves as an indication of the overall muscular power, which declines with age (49). Research has demonstrated that the muscle strength of individuals aged 50 and above declines by 12 to 14 percent every ten years (50). Moreover, lower muscle strength has been associated with a higher risk of having impaired daily living capabilities (26, 47, 48). A recent study discovered that HGS asymmetry and weakness were independently correlated with functional disability and functional dependency among middle-aged and older Chinese adults, suggesting that higher muscle strength endows individuals with a protective reserve against the development of dependence in BADL and IADL (47).

Simultaneously, we discovered that functional limitation was inversely associated with subsequent depressive symptoms, which aligns with the findings of previous studies (32, 51, 52). This implies that limitation in ADL is a risk determinant for depressive symptoms. Functional limitation curtails an individual’s ability to perform essential or fundamental daily tasks, including housework, cooking, shopping, and even basic self-care activities such as dressing and eating. Such limitations lead to restrictions in social participation, mobility, and self-care (53). With the deterioration of health, it may trigger latent physical, cognitive, or emotional impairments that can give rise to depressive symptoms (54).

Most importantly, following the adjustment for covariates, this research validated that functional limitation mediates the connection between diminished HGS and depressive symptoms through the application of the activity restriction model. This mediation effect accounted for 18.9% of the association. A plausible explanation, from the vantage point of stress theory, is that functional limitation can be considered as a chronic stressful event that induces a progressive loss of daily living abilities among individuals. Such a loss may subsequently contribute to an elevated hazard of depressive symptoms (54). As middle-aged and older individuals age, they commonly experience a gradual attenuation of muscle strength, which precipitates a successive decline in physical function (48). Consequently, they might become incapacitated in performing more complex self-care behaviors, including cooking, making phone calls, and managing finances. When the decline in muscle strength extends to the most rudimentary self-care activities, like eating, dressing, and bathing, they may lose the capacity to independently manage their daily lives and thus rely on external assistance (31). This situation engenders a sense of low self-worth and futility (32, 54). Simultaneously, the reduction in self-care ability leads to a diminution in social activities. Middle-aged and older adult individuals with limited social participation possess attenuated social roles, which gives rise to a perception of detachment from society and a sense of social isolation. These factors can potentially trigger the development of depressive symptoms (55).

It is crucial to note that the role of other potential unmeasured confounding factors should be considered. For example, cognitive impairment is believed to be independently related to sarcopenia and depressive symptoms (56, 57), which could account for the remaining variance. Moreover, studies have indicated that older adult individuals with reduced HGS are more prone to cognitive impairment (58, 59), and cognitive impairment (in terms of executive functioning, memory, and attention) is prevalent among patients with depression (60). Hence, the impact of cognitive function domains on the relationship between HGS and depressive symptoms demands further investigation. In addition, age-related chronic inflammation and oxidative stress are regarded as shared mechanisms between sarcopenia and depressive symptoms. Physical activity or resistance training can assist in alleviating depressive symptoms by augmenting muscle strength and reducing systemic inflammation (61). Therefore, future research is essential to explore how diverse factors mediate the relationship between HGS and depressive symptoms.

There are several limitations in this research. Firstly, the results do not establish causation because this study was, in fact, observational. Secondly, the measurement of depressive symptoms relied on retrospective self-reports by respondents, thereby carrying a potential risk of recall bias. Moreover, selection bias manifests because we excluded many participants who lacked major variables. Thirdly, some residual confounders from unmeasured factors such as cognitive impairment and inflammatory cytokines could not be precluded, notwithstanding the control of many potential covariates. Finally, due to data limitations, only a three-year follow-up period from 2015 to 2018 was examined. Future research should contemplate longer tracking durations and explore possible mechanisms.

Despite the inherent limitations of this study, it yields valuable insights with significant implications for clinical practice. Our findings indicate that strong muscle strength could improve the degree of functional limitation and decrease the probability of manifesting depressive symptoms in the future. This would imply that we can further improve physical function by enhancing muscle strength to lower the incidence of depressive symptoms among middle-aged and older individuals. Accordingly, healthcare providers in community hospitals have a responsibility to strengthen screening procedures for muscle strength and daily activity capabilities in the older adult population. The aim is to identify individuals with diminished HGS and impaired ADL performance. Subsequently, timely and effective intervention strategies, such as increasing physical activity, implementing progressive resistance training programs, and formulating personalized nutritional plans (with a specific focus on vitamin D supplementation), should be employed to prevent the onset of depressive symptoms.

## 5 Conclusion

The present study verified that functional limitation mediated the relationship between HGS and subsequent depressive symptoms in middle-aged and older adults via the activity restriction model. This discovery emphasizes the necessity of promoting HGS screening among middle-aged and older adults, notably those with initially low HGS, and implementing targeted interventions to enhance muscle strength. Such measures are crucial for delaying functional limitation and preventing the occurrence of depressive symptoms.

## Data Availability

Publicly available datasets were analyzed in this study. This data can be found at: https://charls.pku.edu.cn/.
