# Supplementary material for: Longitudinal association between handgrip strength and depressive symptoms in middle-aged and older Chinese adults: mediating role of functional limitation
Source: Front Public Health. 2025 Feb 18;13:1496641. doi: 10.3389/fpubh.2025.1496641 (PMC11876040; doi:10.3389/fpubh.2025.1496641)
Supplement: Supplementary file 1 [file Supplementary_file_1.docx]

| **Supplementary Table S1.** The mediating effect of functional limitation on the relationship between handgrip strength at baseline and depressive symptoms at follow-up in middle-aged and elderly people (N = 4827) | | | | | | | | | |
| --- | --- | --- | --- | --- | --- | --- | --- | --- | --- |
|  | Model 1 | | | | Model 2 | | | | |
|  | B | SE | β | P value | B | | SE | β | P value |
| Handgrip strength |  |  |  |  | |  |  |  |  |
| **Quartile 4** |  |  |  |  | |  |  |  |  |
| **Quartile 1** | 1.78 | 0.30 | 0.13 | **<0.001** | | 1.46 | 0.30 | 0.10 | **<0.001** |
| **Quartile 2** | 0.82 | 0.28 | 0.06 | **0.003** | | 0.74 | 0.27 | 0.05 | **0.006** |
| **Quartile 3** | 0.60 | 0.25 | 0.04 | **0.016** | | 0.53 | 0.24 | 0.04 | **0.029** |
| Gender |  |  |  |  | |  |  |  |  |
| Male |  |  |  |  | |  |  |  |  |
| Female | 1.09 | 0.30 | 0.09 | **<0.001** | | 1.00 | 0.29 | 0.08 | **0.001** |
| Age | -0.05 | 0.01 | -0.07 | **<0.001** | | -0.06 | 0.01 | -0.10 | **<0.001** |
| Education |  |  |  |  | |  |  |  |  |
| Lower than primary school |  |  |  |  | |  |  |  |  |
| Middle school | -0.98 | 0.22 | -0.07 | **<0.001** | | -0.76 | 0.22 | -0.05 | **<0.001** |
| High school or higher | -1.61 | 0.29 | -0.08 | **0.001** | | -1.33 | 0.28 | -0.07 | **<0.001** |
| Marital status |  |  |  |  | |  |  |  |  |
| Married and cohabiting |  |  |  |  | |  |  |  |  |
| Married and separated | -4.54 | 5.79 | -0.16 | 0.433 | | -1.40 | 5.71 | -0.05 | 0.807 |
| Divorced or Widowed | -3.73 | 5.78 | -0.18 | 0.520 | | -0.66 | 5.70 | -0.03 | 0.908 |
| **Residence** |  |  |  |  | |  |  |  |  |
| Rural village |  |  |  |  | |  |  |  |  |
| Urban community | 1.10 | 0.18 | 0.09 | **<0.001** | | 0.92 | 0.18 | 0.07 | **<0.001** |
| Working status |  |  |  |  | |  |  |  |  |
| Working |  |  |  |  | |  |  |  |  |
| Retired | -0.10 | 0.22 | -0.01 | 0.660 | | -0.20 | 0.21 | -0.01 | 0.348 |
| Smoking status |  |  |  |  | |  |  |  |  |
| Never |  |  |  |  | |  |  |  |  |
| Former | 0.08 | 0.32 | 0.01 | 0.793 | | 0.09 | 0.31 | 0.01 | 0.776 |
| Current | 0.16 | 0.28 | 0.01 | 0.575 | | 0.15 | 0.27 | 0.01 | 0.593 |
| Drinking status |  |  |  |  | |  |  |  |  |
| Never |  |  |  |  | |  |  |  |  |
| Former | -0.33 | 0.31 | -0.02 | 0.275 | | -0.42 | 0.30 | -0.02 | 0.163 |
| Current | -0.40 | 0.21 | -0.03 | 0.052 | | -0.39 | 0.21 | -0.03 | 0.058 |
| Physical activity |  |  |  |  | |  |  |  |  |
| Moderate |  |  |  |  | |  |  |  |  |
| None | -0.11 | 0.32 | -0.01 | 0.738 | | -0.19 | 0.31 | -0.01 | 0.533 |
| Light | -0.16 | 0.21 | -0.01 | 0.435 | | -0.29 | 0.21 | -0.02 | 0.162 |
| Vigorous | 0.57 | 0.22 | 0.04 | **0.009** | | 0.46 | 0.22 | 0.03 | **0.033** |
| Number of chronic diseases |  |  |  |  | |  |  |  |  |
| 0 |  |  |  |  | |  |  |  |  |
| 1 | 0.78 | 0.22 | 0.06 | **<0.001** | | 0.69 | 0.22 | 0.05 | **0.001** |
| 2 | 1.62 | 0.24 | 0.11 | **<0.001** | | 1.49 | 0.24 | 0.10 | **<0.001** |
| ≥3 | 2.58 | 0.25 | 0.17 | **<0.001** | | 2.21 | 0.25 | 0.15 | **<0.001** |
| **Body mass index** |  |  |  |  | |  |  |  |  |
| Normal |  |  |  |  | |  |  |  |  |
| Underweight | 1.01 | 0.42 | 0.03 | **0.016** | | 0.78 | 0.41 | 0.03 | 0.060 |
| Overweight | -0.89 | 0.20 | -0.07 | **<0.001** | | -0.92 | 0.19 | -0.07 | **<0.001** |
| Obesity | -1.15 | 0.28 | -0.07 | **<0.001** | | -1.18 | 0.27 | -0.07 | **<0.001** |
| Functional limitation |  |  |  |  | |  |  |  |  |
| No limitation |  |  |  |  | |  |  |  |  |
| BADL limitation |  |  |  |  | | 1.85 | 0.32 | 0.08 | **<0.001** |
| IADL limitation |  |  |  |  | | 1.15 | 0.27 | 0.06 | **<0.001** |
| IADL and ADL limitations |  |  |  |  | | 3.85 | 0.33 | 0.17 | **<0.001** |
| F | 19.67 |  |  |  | | 23.71 |  |  |  |
| Adjusted R-squared | 0.088 |  |  |  | | 0.116 |  |  |  |

| **Supplementary Table S2** Mediating effect of functional limitation on handgrip strength and depressive symptoms | | | | | | |
| --- | --- | --- | --- | --- | --- | --- |
|  | **Effect** | **SE** | ***t*** | ***P*** | **95%CI** | |
|  |  |  |  |  | Lower Confidence Interval | Upper Confidence Interval |
| Total effect | -4.878 | 0.755 | -6.463 | <0.001 | -6.358 | -3.398 |
| Direct effect | -4.102 | 0.779 | -5.483 | <0.001 | -5.629 | -2.574 |
| Indirect effect | -0.776 | 0.142^a^ | - | <0.001 | -1.054^b^ | -0.498^c^ |
| SE, Standard Error; ^a^Bootstrap standard error. ^b^BootLow Confidence Interval. ^c^BootUpper Confidence Interval. | | | | | | |


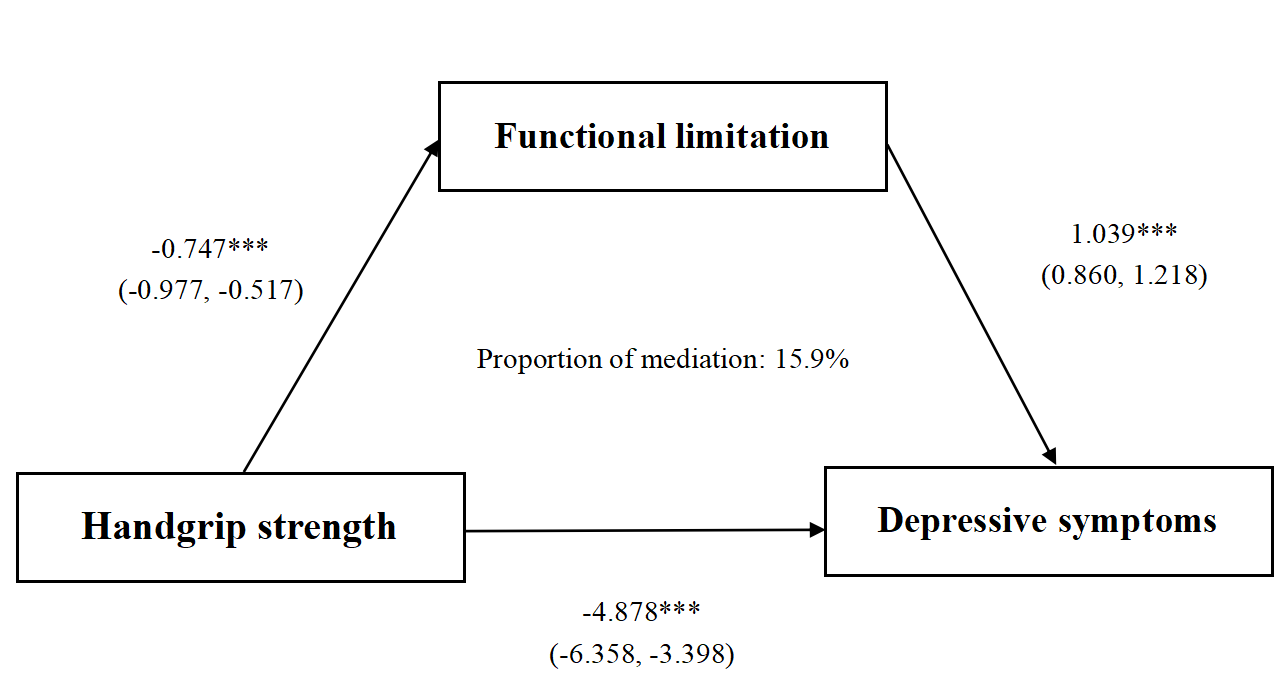


**Supplementary Figure S1**. Path diagram of the association between functional limitation and depressive symptoms as a mediator.

Notes:* * * *P*<0.001. Gender, age, education, marital status, residence, drinking status, smoking status, chronic disease, BMI, physical activity and working status were controlled.
